# Projections of future burden of pharmacologically treated type 2 diabetes and associated life expectancies by income in Finland: a multi-state modeling study

**DOI:** 10.3389/fpubh.2023.1141452

**Published:** 2023-05-26

**Authors:** Maria Guzman-Castillo, Kaarina Korhonen, Michael Murphy, Pekka Martikainen

**Affiliations:** ^1^Population Research Unit, Faculty of Social Sciences, University of Helsinki, Helsinki, Finland; ^2^Department of Social Policy, London School of Economics and Political Science, London, United Kingdom

**Keywords:** type 2 diabetes, income, socioeconomic data, forecast, model, simulation modeling, life expectancies

## Abstract

The burden of type 2 diabetes (T2D) differs between socioeconomic groups. The present study combines ongoing and plausible trends in T2D incidence and survival by income to forecast future trends in cases of T2D and life expectancy with and without T2D up to year 2040. Using Finnish total population data for those aged 30 years on T2D medication and mortality in 1995–2018, we developed and validated a multi-state life table model using age-, gender-, income- and calendar year-specific transition probabilities. We present scenarios based on constant and declining T2D incidence and on the effect of increasing and decreasing obesity on T2D incidence and mortality states up to 2040. With constant T2D incidence at 2019-level, the number of people living with T2D would increase by about 26% between 2020 and 2040. The lowest income group could expect more rapid increases in the number with T2D compared to the highest income group (30% vs. 23% respectively). If the incidence of T2D continues the recent declining trend, we predict about 14% fewer cases. However, if obesity increases two-fold, we predict 15% additional T2D cases. Unless, we reduce the obesity-related excess risk, the number of years lived without T2D could decrease up to 6 years for men in the lowest income group. Under all plausible scenarios, the burden of T2D is set to increase and it will be unequally distributed among socioeconomic groups. An increasing proportion of life expectancy will be spent with T2D.

## 1. Introduction

By 2021, the global number of people living with diabetes had reached 540 million, and this figure is expected to increase almost 50% by 2045 (1). In 2016, diabetes was directly responsible for 1.5 million deaths. Diabetes-related diseases such as cardiovascular disease (CVD) caused 2.2 million additional deaths. Most of these deaths are considered premature. Type 2 diabetes (T2D) accounts for around 90% of the burden of diabetes. T2D is highly prevalent in people aged 65 and older (2). Older people with T2D have higher rates of premature death, functional disability, premature frailty, cardiovascular disease and dementia than do those without T2D (3, 4). Thus, the projected future increase—if actualized—will imminently affect the quality of life of senior citizens and increase costs related to health and social care provision. A better understanding of the past trends and future prospects of T2D is urgently needed.

Past increases in the number of people with T2D have been attributed to behavioral change, improvements in survival of T2D patients and population aging (2). Age-specific incidence of T2D has consistently decreased in many countries, suggesting that efforts to curb the diabetes epidemic have been effective (5). However, increasing obesity worldwide has been identified as a significant threat to continued positive developments. Nevertheless, detailed analysis of the effects of changing obesity rates to future changes in incidence, survival, and the burden of T2D are not available.

T2D disproportionately affects the more disadvantaged in society, at least partly due to socioeconomic gradients in the prevalence of smoking, unhealthy diet (e.g., sugar consumption), obesity, physical inactivity, and hypertension (6, 7). An increased risk of diabetes-related mortality for the lowest income quintiles has been consistently reported (8, 9). However, little is known about how changes in T2D incidence and survival have and will contribute to social inequalities in T2D burden and life expectancy with and without T2D.

Governments, policy makers and clinicians need detailed understanding of the past trends and reliable forecasts of the future burden of T2D. Thus far, previous forecasts have not considered the effect of health inequalities in T2D, past trends of its incidence and mortality, nor the contribution of obesity over time. In this study, we set out to forecast future trends in the burden of pharmacologically managed T2D in Finland up to year 2040, simultaneously accounting for ongoing trends in incidence and survival by income. In addition, we also explore the potential effect of future obesity trends and estimate life expectancy with and without T2D.

## 2. Materials and methods

### 2.1. Study population and sample

We used population register data covering all Finnish residents aged 30 years or older in any year between 1995 and 2018. Statistics Finland linked the data with annually updated sociodemographic information, mortality follow-up and medication reimbursement register using personal identification codes assigned to all permanent residents. The full analytic sample consisted of 4,887,718 individuals.

### 2.2. Case ascertainment

We identified those with pharmacologically treated T2D using the medication reimbursement register of the Social Insurance Institution of Finland. Individuals were classified as having T2D if at least one of the following criteria was met: (1) they were granted a right to special reimbursement for diabetes medication expenses with T2D diagnosis (ICD-10 codes E11; ICD-9250?A), (2) they had state-reimbursed purchases of medication that are used to increase the secretion of pancreatic insulin (Anatomical Therapeutic Chemical (ATC) codes: A10BB, A10BH, A10BX02, A10BX03, A10BX04), (3) the number of years with any medication (A10B) purchases exceeded the number of years with insulin purchases, (4) the number of years with any medication (A10B) purchases equaled that of insulin purchases, and the age at first purchase was 40+ years, or (5) they were granted a special reimbursement for diabetes medication expenses with an unspecified diabetes diagnosis at the age of 40 years or over. The year of T2D incidence was defined according to the earliest entry in the reimbursement register. This identification of T2D has been previously used and verified (10).

### 2.3. Socioeconomic position

We used household income as the measure of socioeconomic position, comprising the disposable annual income of all household members, including salaries, entrepreneurial and property income, transfers received and taxes paid. The information was obtained from the Finnish Tax Administration and the Social Insurance Institution of Finland. We adjusted for household composition using the OECD-modified equivalence scale (11). Income quintiles were formed based on the income distribution in 5-year age strata in each year.

### 2.4. Statistical analyses

We developed and validated a multi-state life table model that simulates transitions of the Finnish population aged 30 years or more through the states of illness (T2D) and mortality. Movements of the population between these states are governed by 1 year age-, gender-, income-, and calendar-year specific probabilities of transition. At each year of iteration a new cohort of 30-year-olds enters the simulation through the disease-free state.

We combined age-specific, gender-specific and income-quintile-specific prevalence data in 1996 from the population register with population estimates from Statistics Finland to populate the states in the model at the start of the simulation. We used projections from the Statistics Finland until 2040 along with the income structure of the population to create the input population vector of 30-year-olds assumed to be disease-free at entry in subsequent years.

Our baseline scenario assumed that the gender, age and income quintile -specific incidence of T2D will remain constant after year 2019 until 2040. In this scenario, we projected strata-specific past trends in mortality in 1995–2018 into the future for both the T2D and non-T2D groups with generalized additive models keeping differentials constant at 2019 levels within income groups.

In view of uncertainty regarding trends in T2D incidence, we assessed three additional scenarios:

- Scenario A: we assumed a continuation of the declining trend in T2D incidence observed since 2011 (Supplementary Figure S1) and projected this trend into the future by gender, age and income quintile.
- Scenario B: we assumed an elimination of the excess risk related to obesity on T2D incidence and mortality. This scenario uses the population attributable fraction (PAF), an epidemiological method that measures the proportion of the disease burden that can be attributed to a specific risk factor exposure in a population. Therefore, we estimated the incidence and mortality not attributable to obesity exposure by multiplying the future transition probabilities to T2D and mortality from T2D in scenario A by 1-PAF.
- Scenario C: we assumed that the contribution of obesity to T2D incidence and mortality would double compared with that observed in 2019. We then recalculated the gender, age, income quintile and calendar year specific future trend of T2D incidence and T2D mortality in scenario A by multiplying the appropriate transition probabilities by 1 + PAF.

We used the model to calculate future trends in the prevalence of T2D and life expectancies at 65 with and without T2D under the scenarios described above. To validate our outputs, we compared our model estimates with observed mortality data from the Human Mortality Database (HMD) for the period 1996–2019. For new and prevalent T2D cases and mortality among the T2D population, we compared our model estimates with those published in the Diabetes in Finland (FinDM) project for the period 2000–2017. Finally, we compared our estimates of life expectancy (LE) at age 65 years with those reported by the HMD for selected years. The Supplementary material provides detailed information about data sources, methods, assumptions and validation.

## 3. Results

The prevalence of T2D in 2019 was slightly higher among men than women, and in the lower income groups (Table 1). However, most of the cases of T2D in lower income groups are women. For example, the proportion of women in the lowest income group is 54% against 39% in the highest income group. The prevalence was highest in the age group 80–90 years, although most of the cases concentrated around ages 60–80 years.

**Table 1:** Number of people free of type 2 diabetes (T2D) and people with T2D in 2019 in Finland (in thousands).

		Non-T2D	T2D
Gender	Men	1,554,000 (87.6%)	219,000 (12.4%)
	Women	1,688,000 (89.3%)	203,000 (10.7%)
Income	Q1(highest income)	656,400 (91.0%)	65,100 (9.0%)
	Q2	649,100 (89.4%)	77,100 (10.6%)
	Q3	655,900 (88.6%)	84,400 (11.4%)
	Q4	644,300 (87.4%)	92,700 (12.6%)
	Q5 (most deprived)	636,400 (86.1%)	102,700 (13.9%)
Age	30–39	693,200 (98.2%)	13,000 (1.8%)
	40–49	630,300 (95.8%)	27,600 (4.2%)
	50–59	664,300 (90.7%)	68,500 (9.3%)
	60–69	606,400 (83.7%)	118,300 (16.3%)
	70–79	417,500 (77.3%)	122,400 (22.7%)
	80–89	189,300 (75.4%)	61,900 (24.6%)
	90+	41,200 (79.8%)	10,400 (20.2%)

Estimates by gender, income quintile and 10-year age bands.

**Figure 1:**
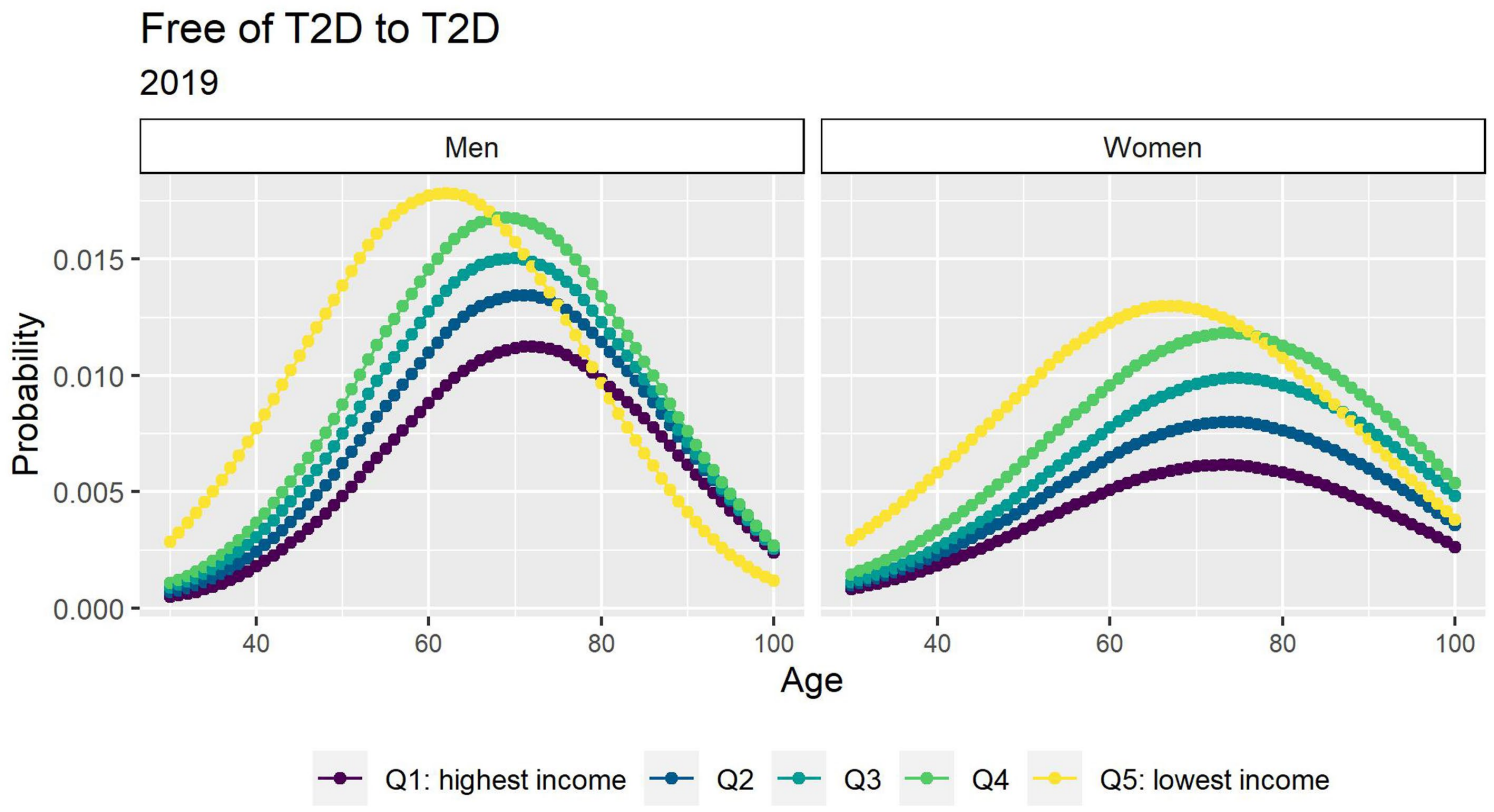
Probabilities of transition between the free of T2D and T2D states in 2019 by income quintile.

**Figure 2:**
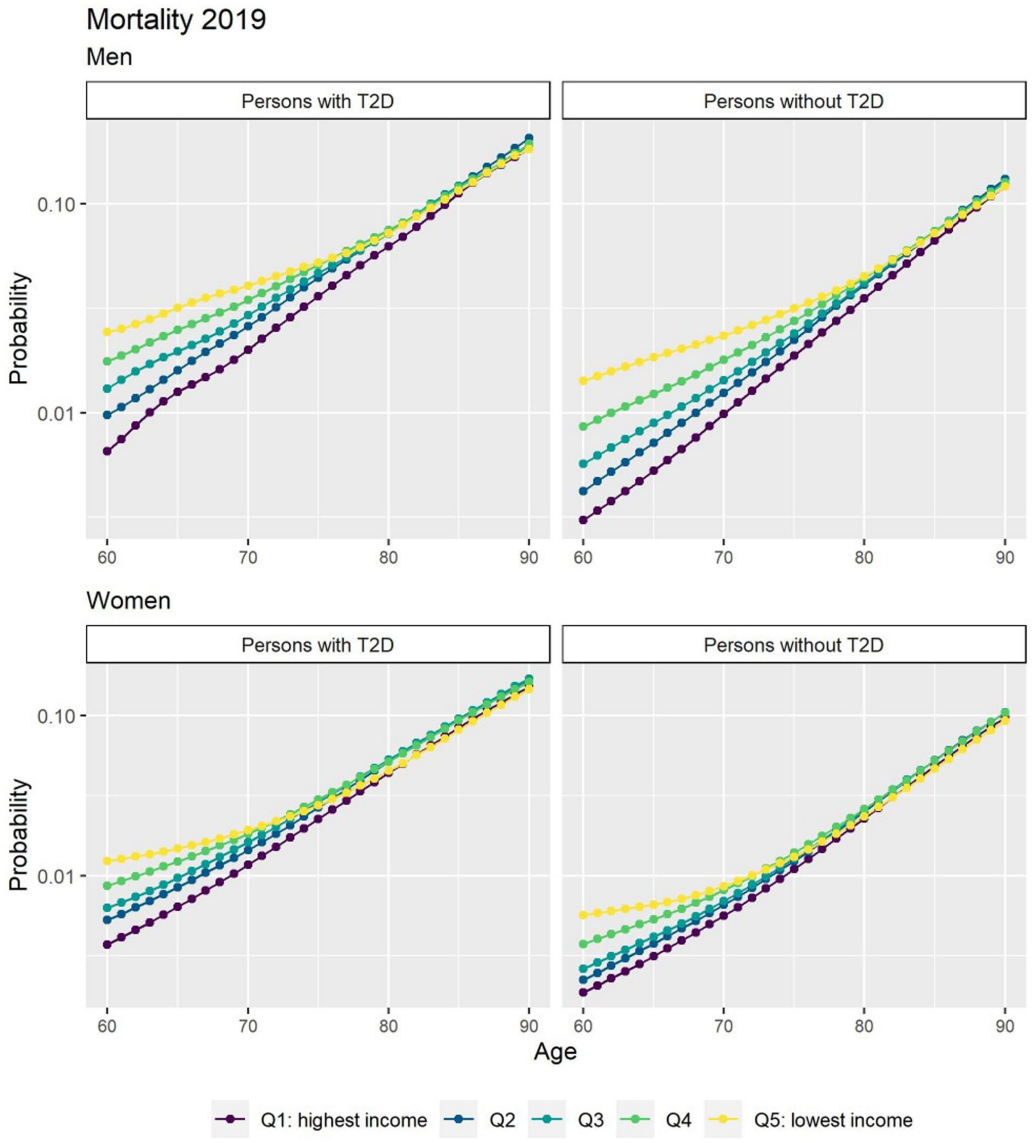
Probabilities of transition to death for people free of T2D and people with T2D in 2019 by income quintile. ^*^The y axis scale has been transformed into a log scale base 10.

Figure 1 depicts the risk of T2D peaked after age 60 years and was higher in men. There was a strong income gradient where the lower income groups in both genders have a higher probability to develop T2D at earlier age. People with T2D had a consistent excess in the risk of death that diminished with age (Figure 2).

If the incidence of T2D remains constant at current levels until 2040 (baseline scenario), the number of people living with T2D will increase by about 26% between 2020 and 2040. By 2040, roughly 538,000 people will be living with T2D, an additional 110,000 cases compared with 2020 (Table 2). The number of people with T2D in the lowest income group will increase by 30%, compared to a 23% increase in the highest income group. Consequently, the gap between the lowest and highest income quintile will increase over time.

**Table 2:** Number of simulated type 2 diabetes (T2D) prevalent cases in 2020, 2030 and 2040 by income quintile (baseline scenario).

	2020	2030	2040	Diff 2020–2030	Diff 2020–2040
All	428,400	495,100	537,900	66,700 (15.6%)	109,500 (25.6%)
Q1: highest income	66,900	76,600	82,100	9,700 (14.5%)	15,200 (22.8%)
Q2	76,400	87,600	94,300	11,200 (14.6%)	17,800 (23.3%)
Q3	84,900	98,200	106,300	13,300 (15.7%)	21,400 (25.2%)
Q4	93,300	10,7200	116,300	13,900 (14.9%)	23,000 (24.7%)
Q5:most deprived	106,900	125,500	138,900	18,600 (17.4%)	32,000 (29.9%)
Difference lowest and highest income quintile	40,000 (59.8%)	48,800 (63.7%)	56,700 (69.1%)		

Despite a shorter total life expectancy (TLE) at age 65 years, those in the lower income groups can expect to live more years with T2D than those with higher incomes (Table 3). In 2020, men and women in the lowest income quintile had a LE with T2D of 4.8 and 5.5 years, compared to 4.2 and 3.4 years among men and women in the highest income quintile.

**Table 3:** Total life expectancy at 65 and life expectancy with and without type 2 diabetes (T2D) in 2019 and 2040 by income quintile (baseline scenario).

	Income quintile	Total life expectancy at 65			Life expectancy without T2D at 65			Life expectancy with T2D at 65			Proportion of life without T2D			2020	2040	Diff 2020–2040	2020	2040	Diff 2020–2040	2020	2040	Diff 2020–2040	2020	2040	Diff 2020–2040
Men	Q1 (least deprived)	20.9	25.0	4.1 (19.6%)	16.7	19.3	2.6 (15.6%)	4.2	5.7	1.5 (35.7%)	79.9%	77.2%	−2.7%
	Q2	19.6	23.8	4.2 (21.4%)	15.2	17.6	2.4 (15.8%)	4.4	6.2	1.8 (40.9%)	77.6%	73.9%	−3.6%
	Q3	19.2	23.5	4.3 (22.4%)	14.5	16.7	2.2 (15.2%)	4.7	6.8	2.1 (44.7%)	75.5%	71.1%	−4.5%
	Q4	18.4	22.9	4.5 (24.5%)	13.5	15.6	2.1 (15.6%)	4.9	7.3	2.4 (49%)	73.4%	68.1%	−5.2%
	Q5 (most deprived)	17.7	22.3	4.6 (26%)	12.9	14.3	1.4 (10.9%)	4.8	8	3.2 (66.7%)	72.9%	64.1%	−8.8%
	Diff Q5 and Q1	−3.2 (−15.3%)	−2.7 (−10.8%)		−3.8 (−22.8%)	−5 (−25.9%)		0.6 (14.3%)	2.3 (40.4%)		−7.0%	−13.1%	
Women	Q1 (least deprived)	23.7	27.5	3.8 (16%)	20.3	23.4	3.1 (15.3%)	3.4	4.1	0.70 (20.6%)	85.7%	85.1%	−0.6%
	Q2	22.9	26.7	3.8 (16.6%)	18.9	21.9	3 (15.9%)	4	4.8	0.80 (20%)	82.5%	82.0%	−0.5%
	Q3	22.6	26.3	3.7 (16.4%)	18.1	20.7	2.6 (14.4%)	4.5	5.6	1.1 (24.4%)	80.1%	78.7%	−1.4%
	Q4	22.2	25.9	3.7 (16.7%)	17.1	19.3	2.2 (12.9%)	5.1	6.6	1.5 (29.4%)	77.0%	74.5%	−2.5%
	Q5 (most deprived)	22.6	26.4	3.8 (16.8%)	17.1	18.5	1.4 (8.2%)	5.5	7.9	2.4 (43.6%)	75.7%	70.1%	−5.6%
	Diff Q5 and Q1	−1.1 (−4.6%)	−1.1 (−4%)		−3.2 (−15.8%)	−4.9 (−20.9%)		2.1 (61.8%)	3.8 (92.7%)		−10.0%	−15.0%	

By 2040, the gradient in TLE at age 65 years by income is expected to slightly narrow in men and remain constant in women. Yet the difference in LE with T2D between socioeconomic groups will further broaden, as the increase in LE with T2D is largest for the lower income group. This group could face an increase of 3.2 years for men and 2.4 years for women in LE with T2D, which is in sharp contrast for the increases of 1.5 and 0.7 years for men and women, respectively, in the highest income group (Table 3). Consequently, the proportion of life spent without T2D will decrease by 9% for men and 7% for women in the lowest income group compared to 3 and 0.3%, respectively, in the highest income group.

### 3.1. Scenarios

**Figure 3:**
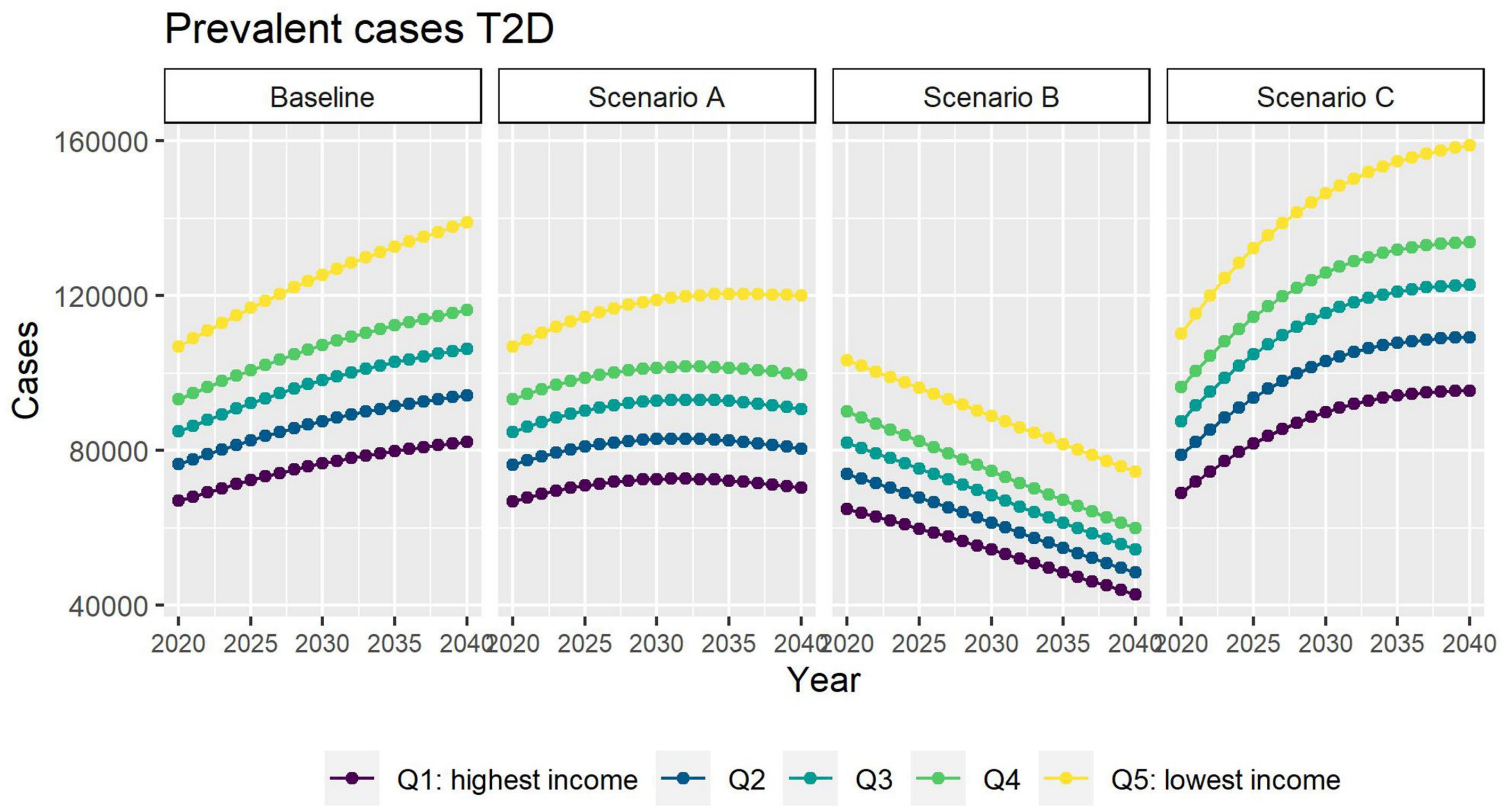
Number of prevalent T2D cases between 2019 and 2040 by income quintile under four different assumptions of T2D incidence trends.

Figure 3 depicts the future number of prevalent T2D cases using alternative assumptions for incidence. If incidence of T2D continues the decline as it has since 2011 (scenario A), roughly 470,000 people will be living with T2D by 2040, approximately 78,000 (14%) fewer cases than the baseline scenario. Our results also suggest that all income quintiles will face a slowdown in the increase of T2D cases.

In scenario B, where we assume no obesity-related excess risk of T2D, we predict 278,000 people living with T2D in 2040 (49% fewer people living with T2D than in the baseline scenario).

In scenario C, we assumed that the contribution of obesity to T2D incidence and mortality will be double compared with that observed in 2019. We predict that by 2040 roughly 630,000 people will be living with T2D. This is about 16% more than in the baseline scenario.

In all scenarios, the gap between the most and highest income groups will continue to widen up to 2040.

**Figure 4:**
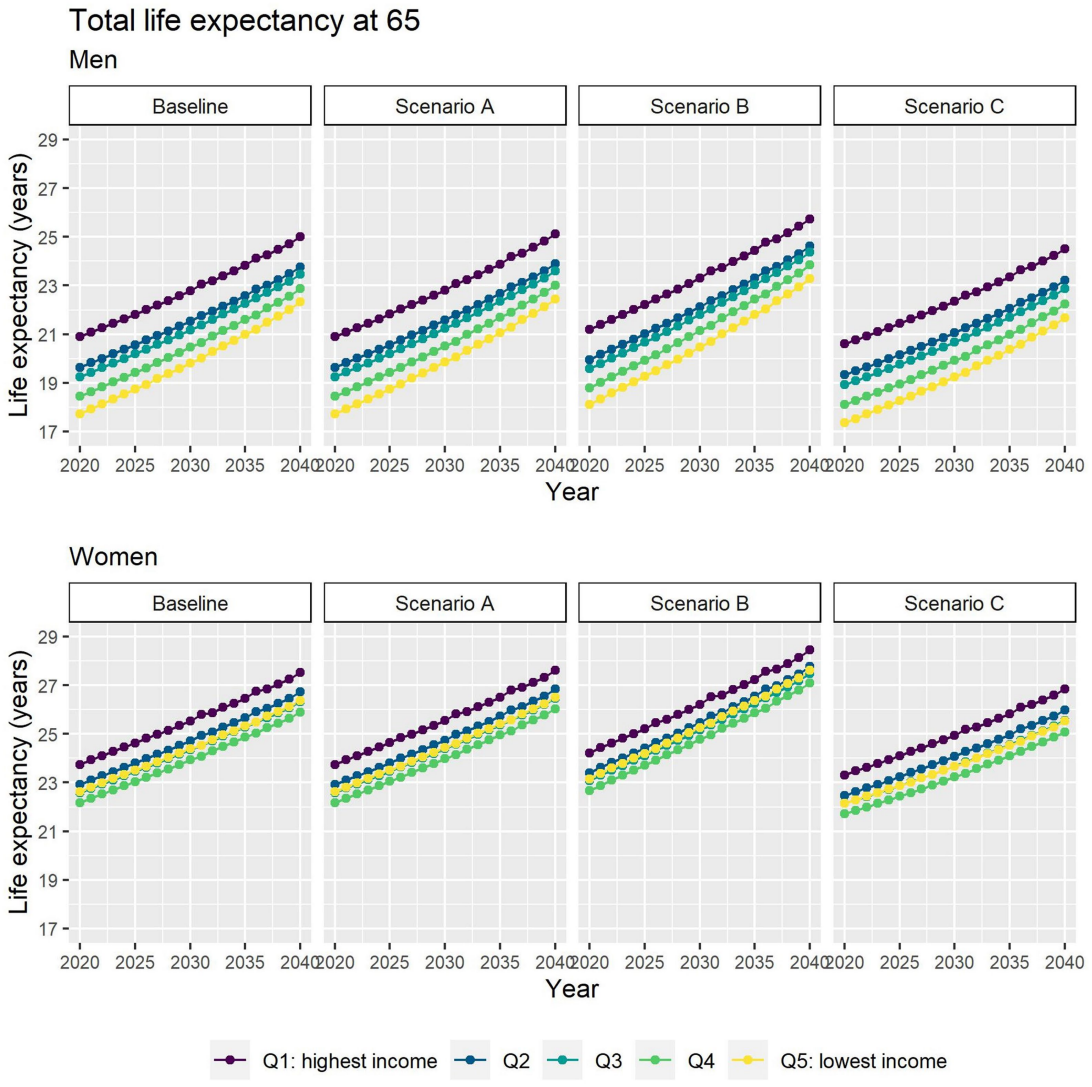
Total life expectancy at 65 for men and women between 2019 and 2040 by income quintile under four different assumptions of T2D incidence trends.

**Figure 5:**
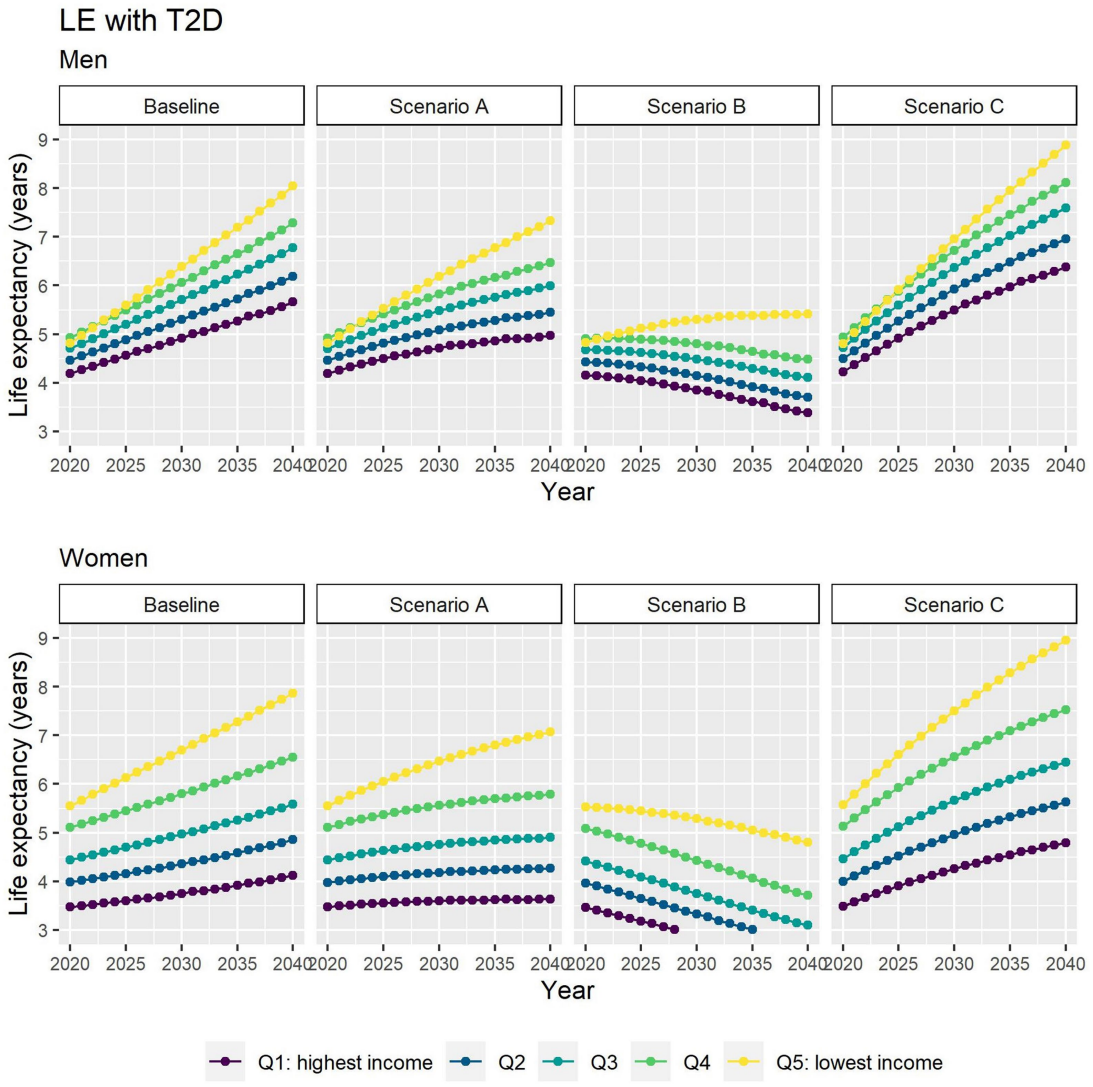
Life expectancy with T2D at 65 for men and women between 2019 and 2040 by income quintile under four different assumptions of T2D incidence trends.

**Figure 6:**
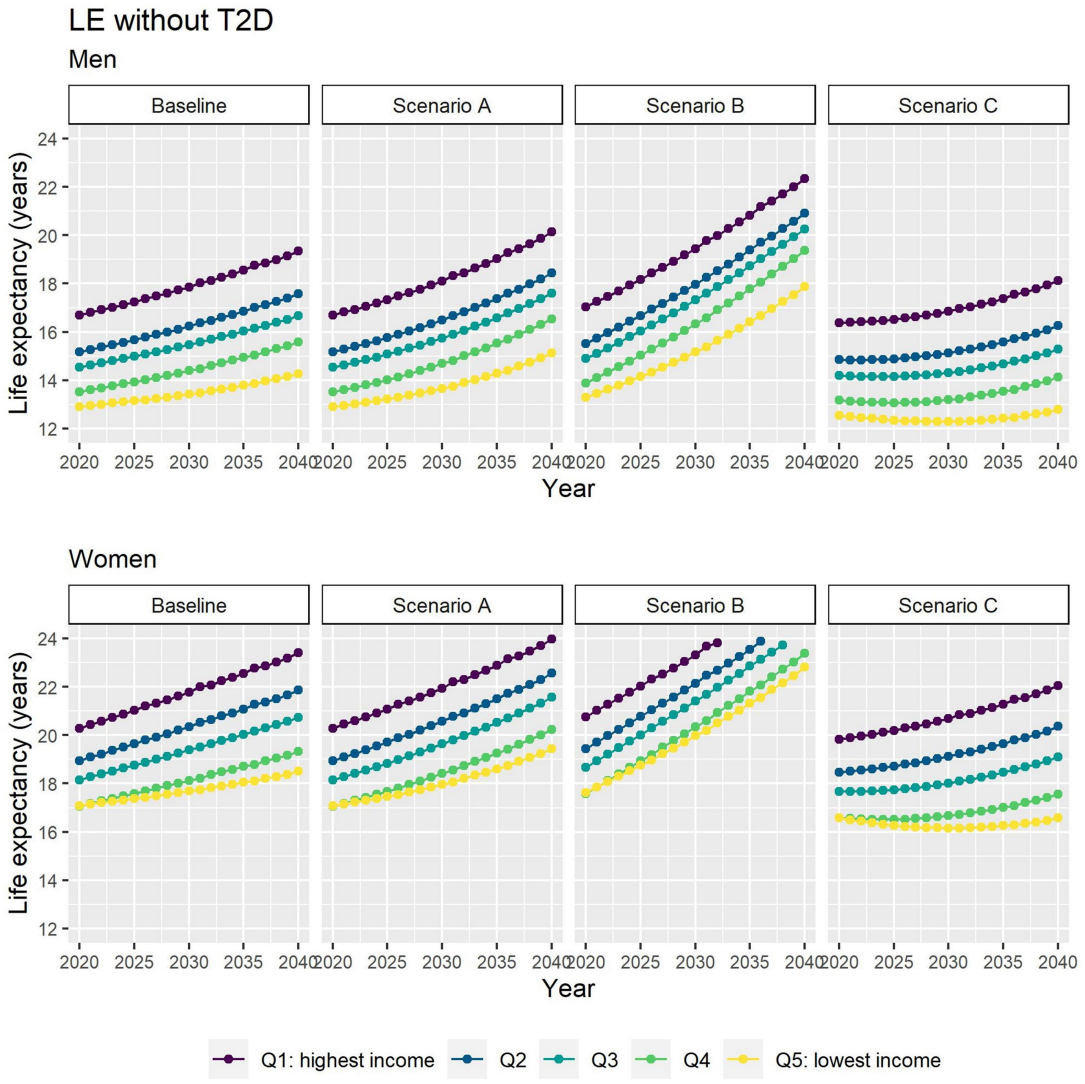
Life expectancy without T2D at 65 for men and women between 2019 and 2040 by income quintile under four different assumptions of T2D incidence trends.

**Figure 7:**
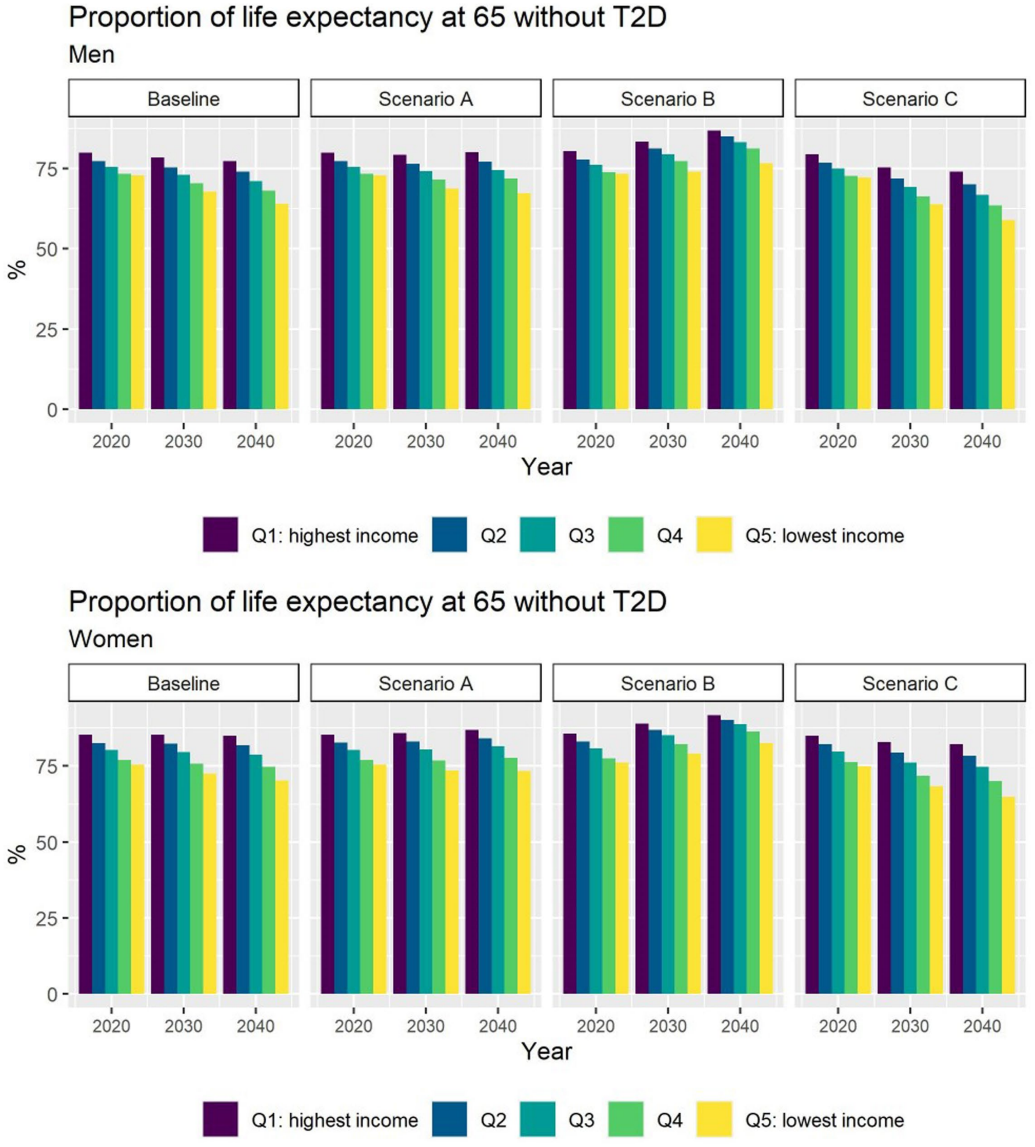
Proportion of life expectancy without T2D at 65 for men and women between 2019 and 2040 by income quintile under four different assumptions of T2D incidence trends.

Different assumptions on future trends of T2D incidence and obesity prevalence have little effect on future estimates of TLE at 65 (Figure 4). LE with T2D will increase for all socioeconomic groups in all scenarios apart from B (Figure 5), while differences between the lowest and highest income groups will continue to widen in all scenarios. LE without T2D is projected to increase in all socioeconomic groups, except for the lowest income group in scenario C (Figure 6). In addition, scenarios A and C forecast widening inequalities in LE without T2D. As a result, the lower income groups can expect to live a smaller proportion of the remaining life at age 65 years free of T2D by 2040, suggesting an expansion of morbidity. The exception would be for scenario B where the elimination of the obesity exposure from the population might increase the number of years spent without T2D (Figure 7).

### 3.2. Validation

We validated our key model outputs against empirical observations. The model estimates of T2D prevalence between 2000 and 2017 were very close to those reported by the FinDM study (Supplementary Figure S2; Supplementary material). However, the model estimates of new T2D cases do not follow the increase in cases between 2007 and 2010 reported by the FinDM study (Supplementary Figure S3). This peak has been reported before and linked to the diabetes prevention and treatment project DEHKO aimed at enhancing diabetes diagnosis (12). Therefore, we excluded these years when estimating T2D incidence trends. Our estimates of TLE at age 65 years for years 1999, 2007, 2011, 2015, and 2019 were very similar to those reported by the HMD (Supplementary Figures S4, S5). Finally, our model provided a good match with the FinDM estimates of mortality among the T2D population (Supplementary Figure S6) and with the number of all-cause deaths reported by the HMD (Supplementary Figures S7, S8).

## 4. Discussion

By 2040, the number of people living with T2D will increase by 26% if the T2D incidence rate continues at the level in 2019; a possible future driven by the aging of the large post WWII birth cohorts and declining mortality. However, if the declining trend in the incidence of T2D observed since 2011 would continue up to 2040, we predict that the future number of T2D cases will only increase by 8%.

Forecasts for T2D and associated life expectancies by income were not available elsewhere. There are however, some studies examining aggregate level estimates. Webber et al. studied non-communicable diseases in 53 WHO European-Region countries and projected for Finland an increase of 170 prevalent diabetes cases per 100,000 between 2020 and 2030 (compared to our estimate of 1,300 per 100,000 for the same period) (13). A multistate model by Fink et al., also projected an increase in the number of cases of T2D among the older adults (75+) in Germany of 67% between 2015 and 2040 (we predicted a 20% increase for the same period) (14). Finally, Tönnies, et al., estimated modest gains in T2D-free life expectancy between 2015 and 2040: 2.6% for women and 4% for men (compared to our estimations of 8% and 9.6%, respectively) (15). However, making comparisons between studies, including ours, is difficult due to different case ascertainment definitions and future trends assumptions. Webber et al. defined T2D as a self-reported diagnosis of T2D and/or serum glucose of at least 7 mmoL/L, whereas Fink et al. and Tönnies et al. used ICD10 codes (E11–E14 and E11 respectively). Webber et al. estimates of T2D prevalence for Finland in 2011 were also below the FinDM report estimates (16). Fink and Weber used incidence data from 2010, potentially missing information on the effect of the most recent trends. Tönnies, et al. used fixed annual declines for incidence and mortality for all the subgroups of the study population, whereas our mortality projections are calendar, age, gender and income specific.

We modeled two contrasting scenarios (B and C) showing the effect of future obesity prevalence trends onT2D incidence and thus on the future number of people living with T2D and associated life expectancies. Our results highlight the urgency of curbing the obesity epidemic as a priority prevention strategy. Although a two-fold increase in obesity seems unlike for Finland, the picture of the obesity epidemic in Finland and elsewhere currently does not look very promising. Recent years have witnessed a deterioration in the quality of diet. According to the FinHealth and FinDiet surveys in 2017, the adult population is far from meeting the official recommendations for consumption of vegetables and fruit, saturated fatty acids and red or processed meat (17, 18). At the same time, levels of exercise did not improve between 2011 and 2017 and even decreased for older women. Moreover, the obesity prevalence has been on the increase in Finland already for two decades (17, 19). Interestingly, this increase does not yet show in the T2D incidence trend of the past 10 years, which may partly relate to a time lag between obesity and the onset of T2D and the possibly countervailing trends in other risk factors.

Our results suggest that the lower income groups not only live shorter lives but also spend a larger proportion of their lives with T2D compared to the more affluent groups. Inequalities in the future burden of T2D and LE with T2D is likely to further widen in the next two decades, regardless of a projected reduction in T2D incidence as the estimates from our different scenarios show. We hypothesize that the historical high burden of T2D in the lowest income group will amplify in the future. This is because even a continued decline in T2D incidence (scenario A) will not be enough to outweigh the effects of mortality decline on increasing T2D prevalence.

### 4.1. Strength and limitations

To our knowledge, this is the first study to estimate the future burden of T2D and the associated LE by socioeconomic position. Our model considers the complex population dynamics of lifespans and ongoing trends in risk, and incorporates the effect of obesity—a major determinant of T2D—to produce credible predictions of future life expectancy with and without T2D.

We used population register data covering all Finnish residents aged 30 years or older avoiding many of the biases which may potentially arise in studies based on limited samples. While many studies have relied on self-reporting of the disease, our definition of diabetes provides a robust clinical representation of T2D status. Furthermore, we cross validated our projections using external data sources and estimates.

Nevertheless, we also acknowledge the limitations of the study. We only considered pharmacologically treated diabetes. This implies that our analyses most likely captures more serious cases of T2D, missing people who follow lifestyle modification guidelines without any medications.

In scenario B and C, we might have underestimated the full effect of future obesity trends on diabetes incidence for income differentials since we used the same initial levels and trends of obesity by income in our scenarios. This was because official estimates of population BMI or prevalence of obesity by income were not available for Finland. Moreover, Salonen et al. only found an income gradient among Finnish middle-aged women in the early 2000s, while the European Health Interview Survey found no income gradient in the obesity prevalence in Finland in 2019 (20, 21). Similarly, we assumed that the relationship between obesity and diabetes is the same across income groups since income-specific relative risks were not available.

Finally, given the nature of our modeling approach, income mobility is not incorporated in the calculation of the transition probabilities. In other words, we assumed individuals stay in the same income quintile. It is not clear to what extent the risk of T2D changes in individuals that change their income or socioeconomic position at these older ages. Similarly, we did not incorporate the duration of T2D on the calculation of the mortality transition probabilities. Previous research has linked a 10-year duration of T2D with a 30% and 60% increased risk of all-cause mortality and CVD mortality, respectively, compared to newly diagnosed cases (22).

### 4.2. Public health implications

As CVD mortality among people with T2D continues to decline, we can expect a further shift in the burden of T2D to non-cardiovascular complications. In particular, T2D may increasingly relate to chronic diseases associated to old age such as dementia, age-related disability and frailty. A recent study from the United Kingdom suggested that a 20% increase in T2D prevalence between 2015 and 2045 would lead to increases of 29% and 47% in new cases of disability and dementia, respectively, among the population aged 65 years and older (23). Therefore, our forecast draws attention to the scale of societal costs associated with T2D in the coming decades. Public and private expenditure on long-term care will need to increase considerably in view of the predicted rise in T2D burden.

Our results highlight the importance to address the widening health inequalities associated to T2D. The Finnish Diabetes Prevention Study (DPS) demonstrated that individualized dietary and physical activity advice carry long-term benefits on T2D risk (24). However, individual-level interventions as such have proven less powerful strategies at reducing inequalities (25). Notably, personalized nutritional counseling and education interventions offer greater benefits for individuals of higher socioeconomic position and thus tend to reproduce inequalities (26). On the other hand, upstream structural interventions such as fiscal measures (taxes, subsidies, or economic incentives) to control obesity seem to be cost saving and more effective at reducing inequalities (27).

In conclusion, our evidence-based forecasting model incorporating possible future trends of T2D incidence, mortality and obesity prevalence predicts that the number of people living with T2D and associated life expectancy with T2D will increase over the next two decades accompanied by an increase in inequalities. This poses a pressing societal challenge and emphasizes the urgent need for policy development aiming at effective prevention.

## Data availability statement

The data analyzed in this study is subject to the following licenses/restrictions: our license from Statistics Finland does not allow the distribution of the data. Requests to access these datasets should be directed to https://www.stat.fi/.

## Author contributions

MG-C, KK, MM, and PM contributed to the study conception and design. KK did the extraction and curation of the data. MG-C designed the methodology, ran the statistical analyses, and drafted the original manuscript. MM provided statistical advice. PM supervised the study. All authors contributed to the article and approved the submitted version.

## Funding

The study was supported by the Academy of Finland (#308247 and # 345219), the European Research Council (grant agreement no 101019329), and the Max Planck–University of Helsinki Center for Social Inequalities in Population Health.

## Conflict of interest

The authors declare that the research was conducted in the absence of any commercial or financial relationships that could be construed as a potential conflict of interest.

## Publisher’s note

All claims expressed in this article are solely those of the authors and do not necessarily represent those of their affiliated organizations, or those of the publisher, the editors and the reviewers. Any product that may be evaluated in this article, or claim that may be made by its manufacturer, is not guaranteed or endorsed by the publisher.

## Supplementary material

The Supplementary material for this article can be found online at: https://www.frontiersin.org/articles/10.3389/fpubh.2023.1141452/full#supplementary-material
