# Supplementary material for: Projections of future burden of pharmacologically treated type 2 diabetes and associated life expectancies by income in Finland: a multi-state modeling study
Source: Front Public Health. 2023 May 26;11:1141452. doi: 10.3389/fpubh.2023.1141452 (PMC10250626; doi:10.3389/fpubh.2023.1141452)
Supplement: Supplementary file 1 [file Table_1.docx]

Supplementary Material

Projections of future burden of pharmacologically treated type 2 diabetes and associated life expectancies by income in Finland: a multi-state modelling study

**Maria Guzman-Castillo, Kaarina Korhonen, Michael Murphy, Pekka Martikainen1**

*** Correspondence:**Maria Guzman-Castillo
maria.guzmancastillo@helsinki.fi

# Overview

We developed a multistate life table model that simulates the transitions of the Finnish population aged 30 or more through states of Type 2 Diabetes (T2D), free of T2D and death. The model is initially populated using age- and gender specific prevalence estimates, with transition probabilities applied at each one-year iteration to predict the number of deaths and prevalence of each state in the next calendar year. The model predicts the future prevalence and mortality of T2D, and life expectancy (LE) with and without T2D.

# Input data

Input data to inform the model include the initial population structure and prevalence of T2D by gender, age and income quintile in 1996 (starting year of the simulation) and transition probabilities between states by gender, age, income quintile and calendar year.

## Missing income data

The full analytic sample consisted of 4 887 718 individuals. From this, 302 138 (6.2%) individuals had missing income information in one or more (but not all) of the follow-up years. To address this, we filled the missing years with their average income from non-missing data across the follow-up.

About 55 970 (1.1%) individuals had missing information of household income in all follow-up years. Most of these individuals were women living in institutional care. Removing them from the analyses would thus have introduced bias in the calculation of mortality and LE. To address the problem, we assigned these individuals to an income quintile while preserving the same age and year-specific distribution of the income groups from the subset of data without missing income.

## Initial prevalence of states

We obtained the numbers of people by gender and single year of age in 1996 from Statistics of Finland. We calculated the proportion of people by age and gender in each income quintile using the population register, pooling years 1996, 1997 and 1998 together, and applied these proportions to the population estimates. Numbers of men and women who reach age 30 and enter the model at each calendar year were obtained from Statistics of Finland population estimates for the years 1996 to 2019 and population projections for the years 2020 to 2040.

The baseline age-, gender- and income-quintile specific prevalence of diabetes was calculated using data from 1995 to 1998 pooled together. We then fitted a Local Polynomial Regression (LOESS) separately for men and women to smooth estimates by single year of age.

## Transition probabilities

The following table summarizes the main assumptions for each transition probabilities for each of the scenarios

| Transition | | Baseline | Scenario A | Scenario B | Scenario C |
| --- | --- | --- | --- | --- | --- |
| From | To |  |  |  |  |
| T2D-free | T2D | Gender-, age- and income quintile-specific incidence of T2D will remain constant after year 2019 until 2040 | We assumed a continuation of the declining trend in gender-, age- and income quintile-specific T2D incidence observed since 2011 until 2040 | We assumed further decline in T2D incidence (from scenario A) due to no obesity-related excess risk of T2D | We assumed a downturn of the declining trend in T2D incidence (from scenario A) due to a two-fold increase in contribution of obesity to T2D incidence from 2019. |
| T2D- free | Death | Gender- and age- specific mortality is projected into the future using generalised additive models BUT keeping income differentials constant at 2019 levels | Same as baseline | Same as baseline | Same as baseline |
| T2D | Death | Gender- and age-specific mortality is projected into the future using generalised additive models BUT keeping income differentials constant at 2019 levels | Same as baseline | We assumed a further decline on the trend in T2D mortality (from baseline) due to no obesity-related excess mortality risk of T2D. Income differences were kept constant | We assumed a downturn of the declining trend in T2D mortality (from baseline) due to a two-fold increase in contribution of obesity to T2D mortality from 2019.  Income differences were kept constant |

Table S 1: main assumptions for each transition probability and scenario

### Transition probability from free of T2D to T2D state

The age-gender and income-quintile probabilities of transition from T2D-free to T2D were obtained by fitting Poisson generalised linear models with exposure as offset and year, age, income, squared term for age and interaction term for age and income quintile as independent variables on subpopulations by gender. The trend in incidence increased from 1996 to around 2009 and then started to decline from 2011 onwards. The peak around years 2007-2009 in the incidence rate has been linked to the Development Programme for the Prevention and Care of Diabetes (DEHKO) aimed at enhancing diabetes diagnosis, as well as the introduction of new treatment recommendations and drug therapies(1,2), therefore these years were excluded from the analysis. Moreover, to fully capture the declining trend after 2011, two Poisson model were fitted using data from 1996 to 2006 and then from 2011 to 2019.

Following the declining trend in incidence of T2D observed in Finland since 2011, we modelled an alternative scenario (scenario A) where the trend is assumed to continue up to year 2040. We used the Poisson model fitted with data from 2010 to 2019 to extrapolate the trend up to 2040 (Figure S1).


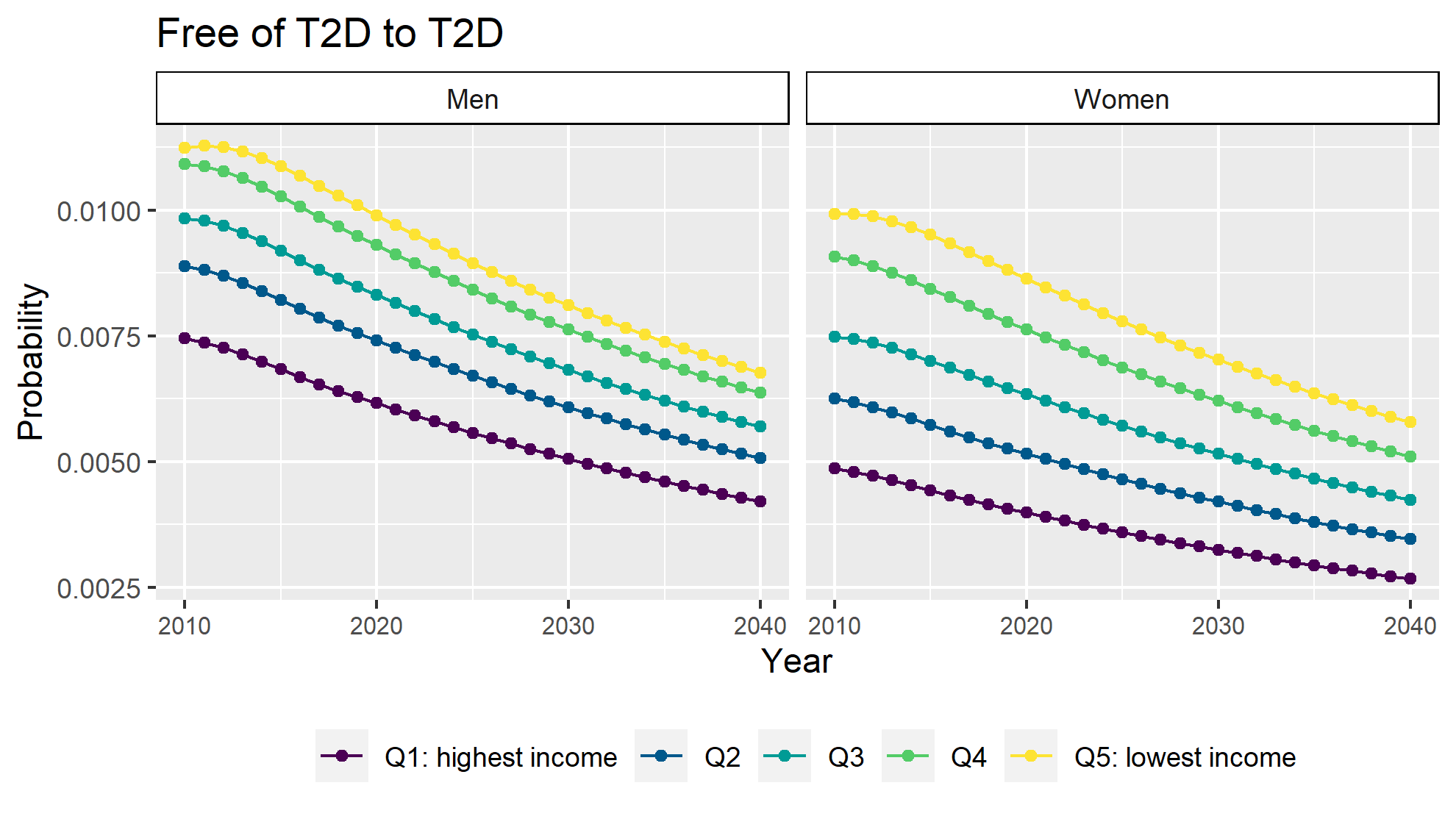


Figure S 1: transition probability from free of Type 2 Diabetes (T2D) to T2D state in 2010-2040 by income quintile and gender under Scenario

## Transition probabilities to death

We projected strata- specific past trends in mortality in 1995-2018 into the future for both the T2D and non-T2D groups with generalised additive models (GAM). GAM are known to estimate more accurately mortality at older ages. The model was from the Poisson family with exposure as offset and smooth functions for year and age by income and linear terms for year and age as independent variables. We fitted separated models on subpopulations by income quintiles.

Despite short term fluctuations observed in our mortality data, there is some evidence suggesting that socioeconomic differentials in life expectancy have changed little in the last 20 years.(3) Therefore to project mortality into the future, we calculate an overall mortality by age and gender until 2040 using GAM and then assuming that the income differentials in mortality observed in 2019 would remain constant until 2040.

## Modelling the effect of future trends in obesity prevalence on T2D incidence and mortality

Scenario B and C assume changes in T2D risk and mortality as result of changes in obesity prevalence. We used the population attributable fraction (PAF), an epidemiological method that measures the proportion of the disease burden that can be attributed to a specific risk factor exposure in a population. In other words what proportion of the disease burden could have been eliminated from the population if nobody was exposed to the risk factor. Its formula is:

$$PAF=\frac{pe\left( RR-1 \right)}{pe\left( RR-1 \right)+1}$$

Where:

$pe$ is the prevalence of the risk factor in the population. For Finland, we used age- and gender-specific obesity prevalence estimates reported by the FinHealth 2017 survey (4).

$RR$ is the relative risk for the disease associated with the risk factor. For T2D incidence we used the hazard ratios reported in (5) and for the transition probabilities to death, those reported in (6).

In scenario B, we assumed an elimination of the excess risk related to obesity on T2D incidence and mortality. Therefore we multiplied the appropriate transition probabilities by $1-PAF$. For example, according to the FinHealth 2017, the prevalence of obesity among men age 50-59 years is 33.9% and the relative risk of obesity for T2D in men is 6.8. This equals to a PAR of 0.66. This means 66% of the new cases of T2D are attributable to obesity. Then, to estimate the incidence not attributable to obesity, we multiplied the transition probability from T2D free to T2D by 0.34

In scenario C, we assumed that the contribution of obesity to T2D incidence and mortality would double compared with that observed in 2019. Using our previous example, we multiply the transition probability from T2D free to T2D by 1.66 ($1+PAF$)

# Validation results

We carried out independent validation (i.e. no information from these sources was used to build the Model) of key model outputs using a graphical approach.


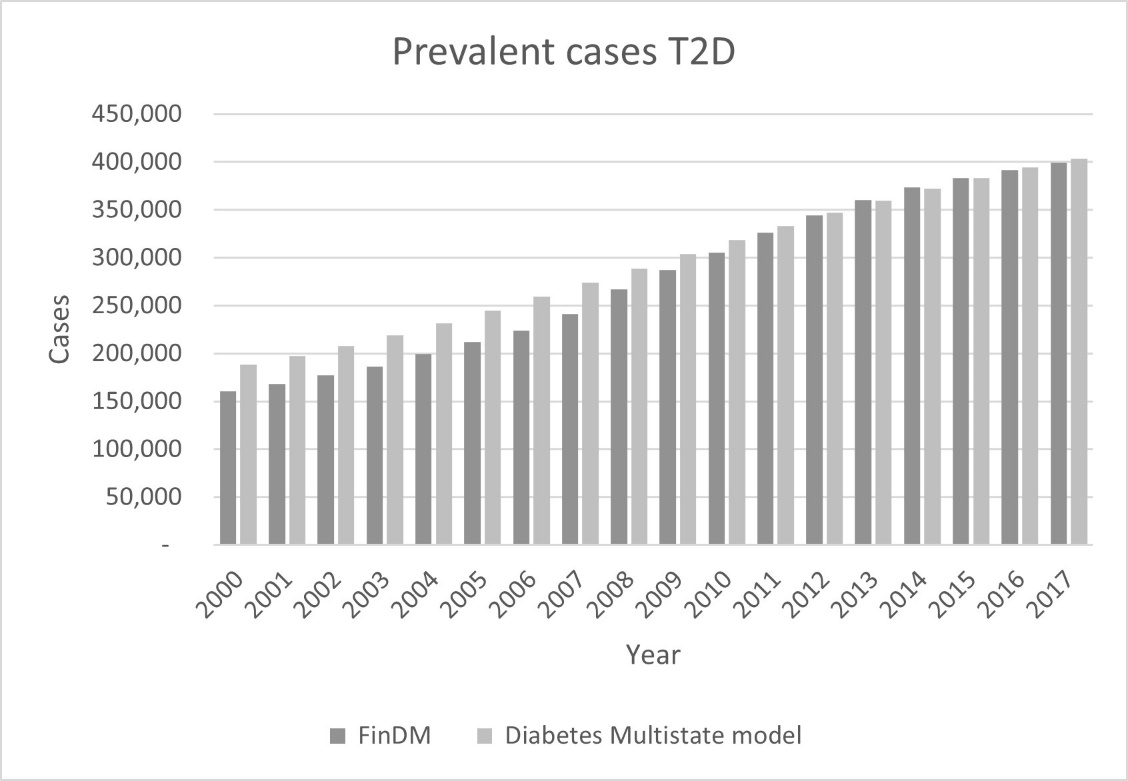


Figure S 2: predicted number of prevalent T2D cases against FinDM 2017 estimates


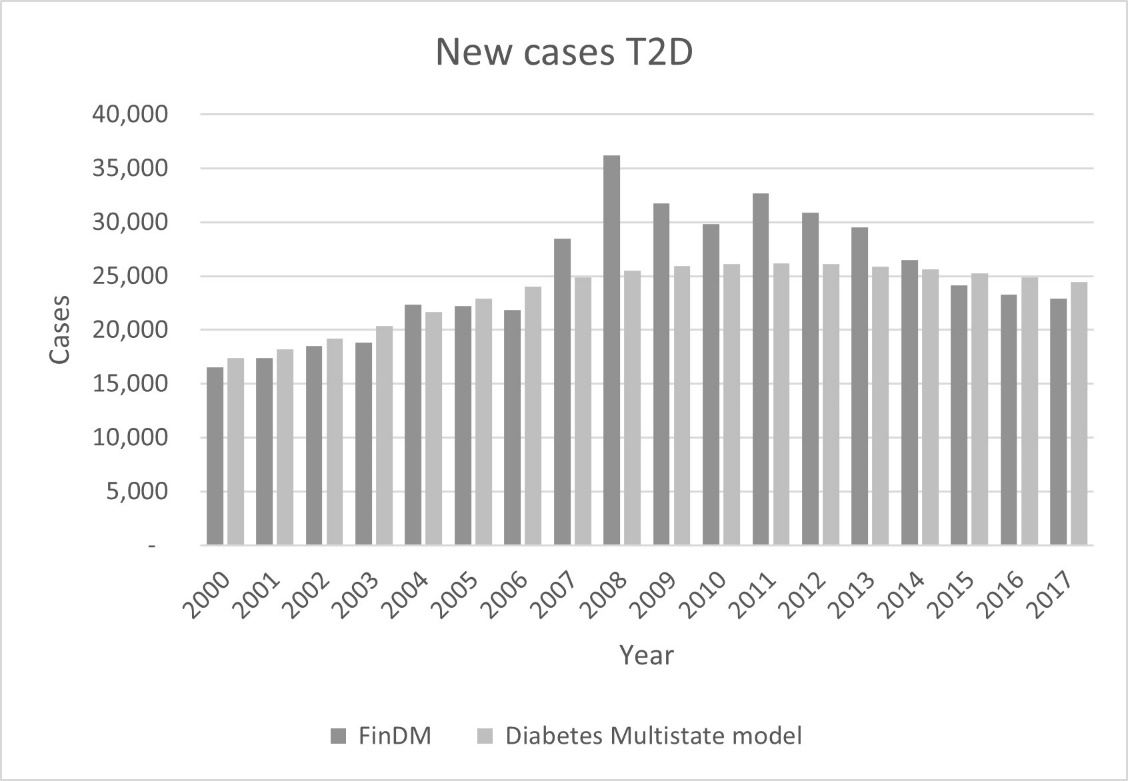


Figure S 3: predicted number of new T2D cases against FinDM 2017 estimates


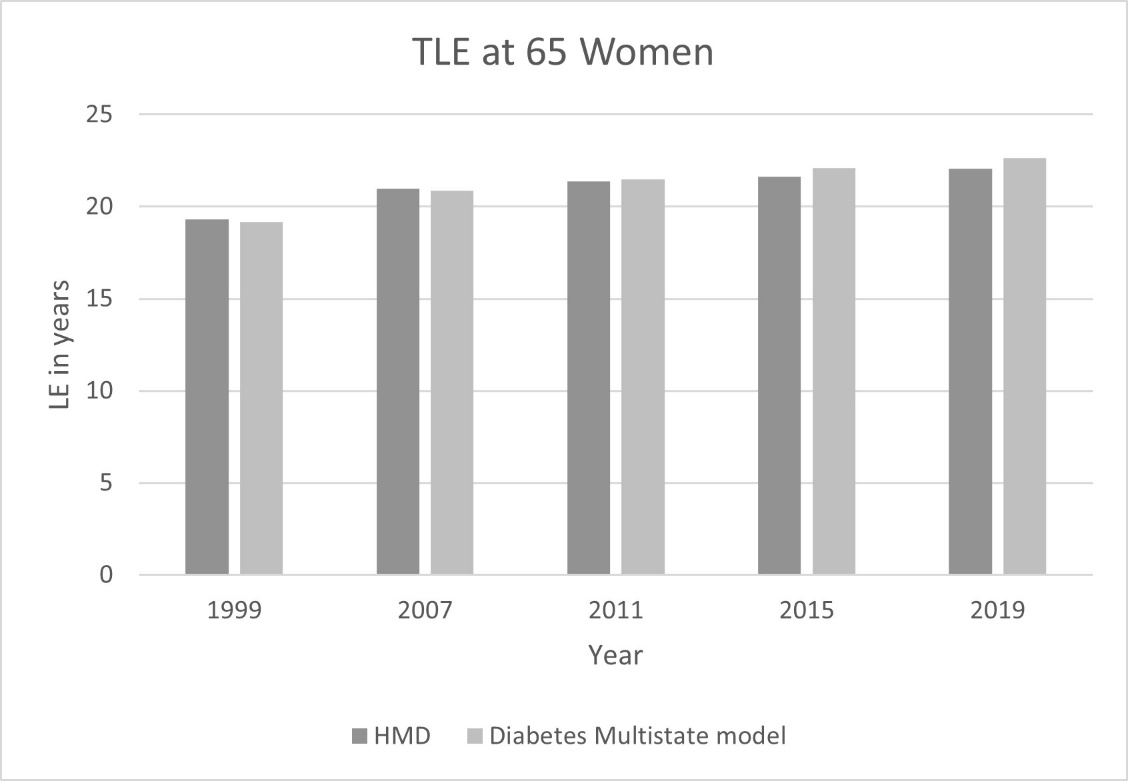


Figure S 4: comparison of estimated total life expectancy (TLE) at age 65 years for women (selected years) against estimates from the FinDM 2017 report.


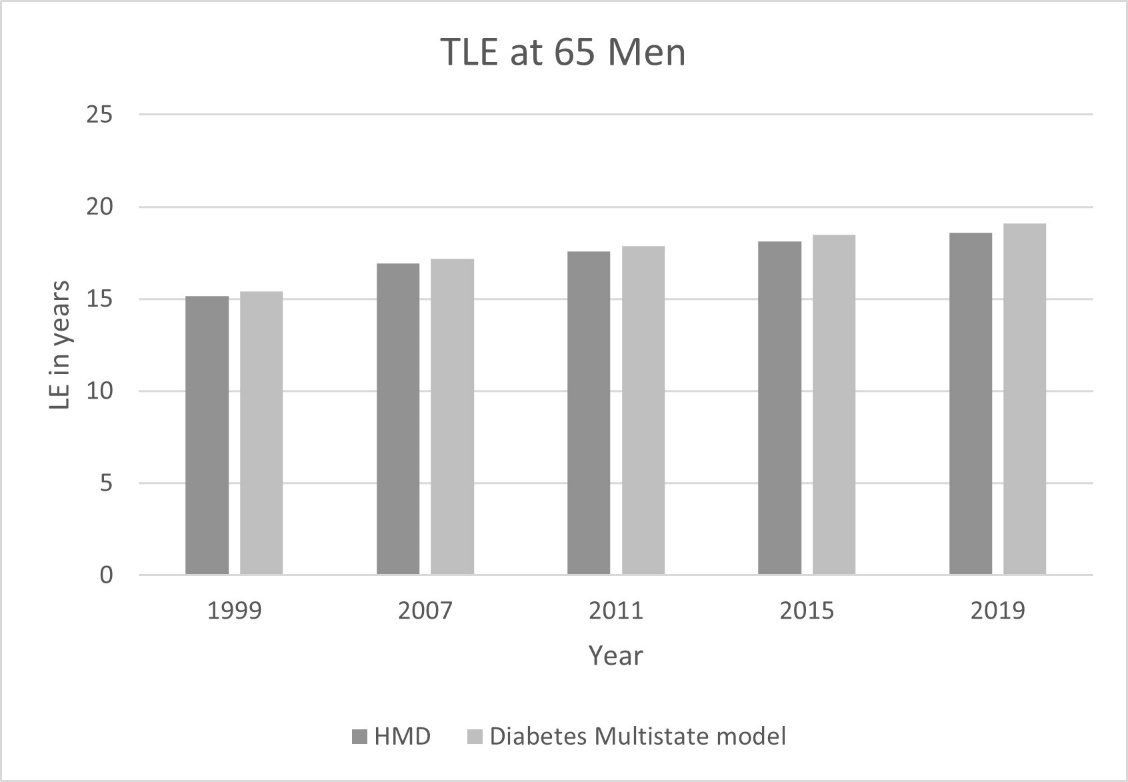


Figure S 5: comparison of estimated total life expectancy (TLE) at age 65 years for men (selected years) against estimates from the FinDM 2017 report.


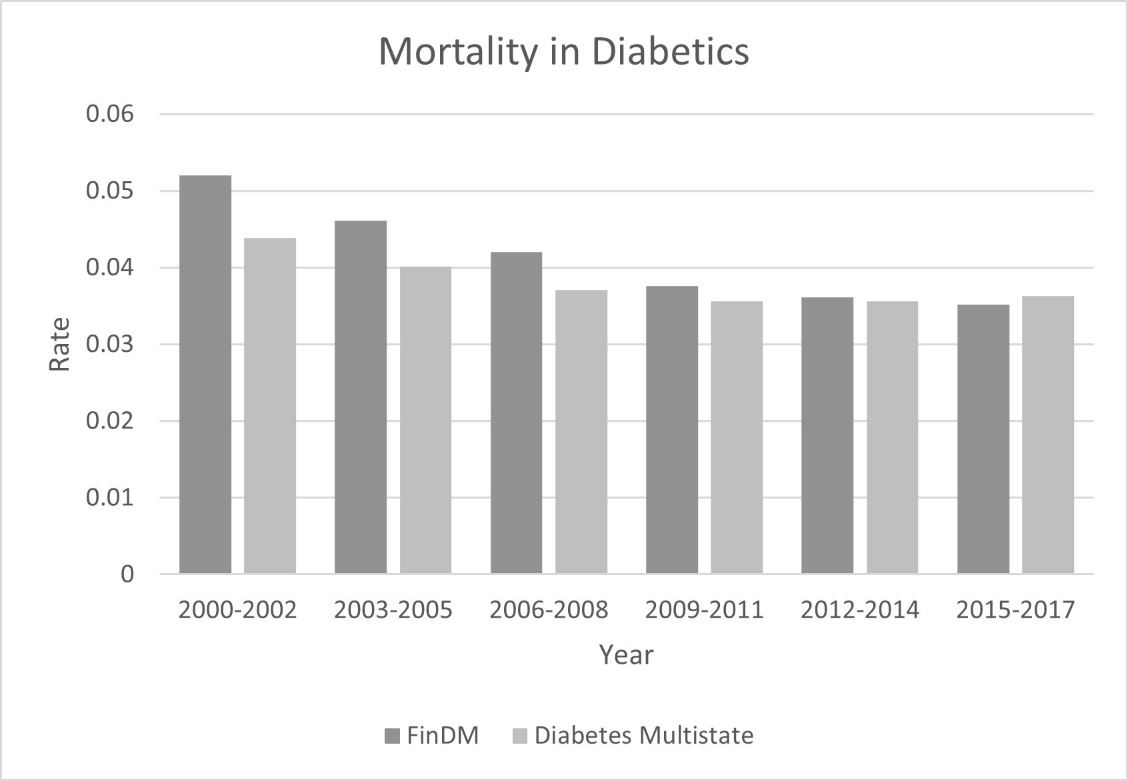


Figure S 6: comparison of estimated mortality rate among the population with T2D against FinDM 2017 estimates


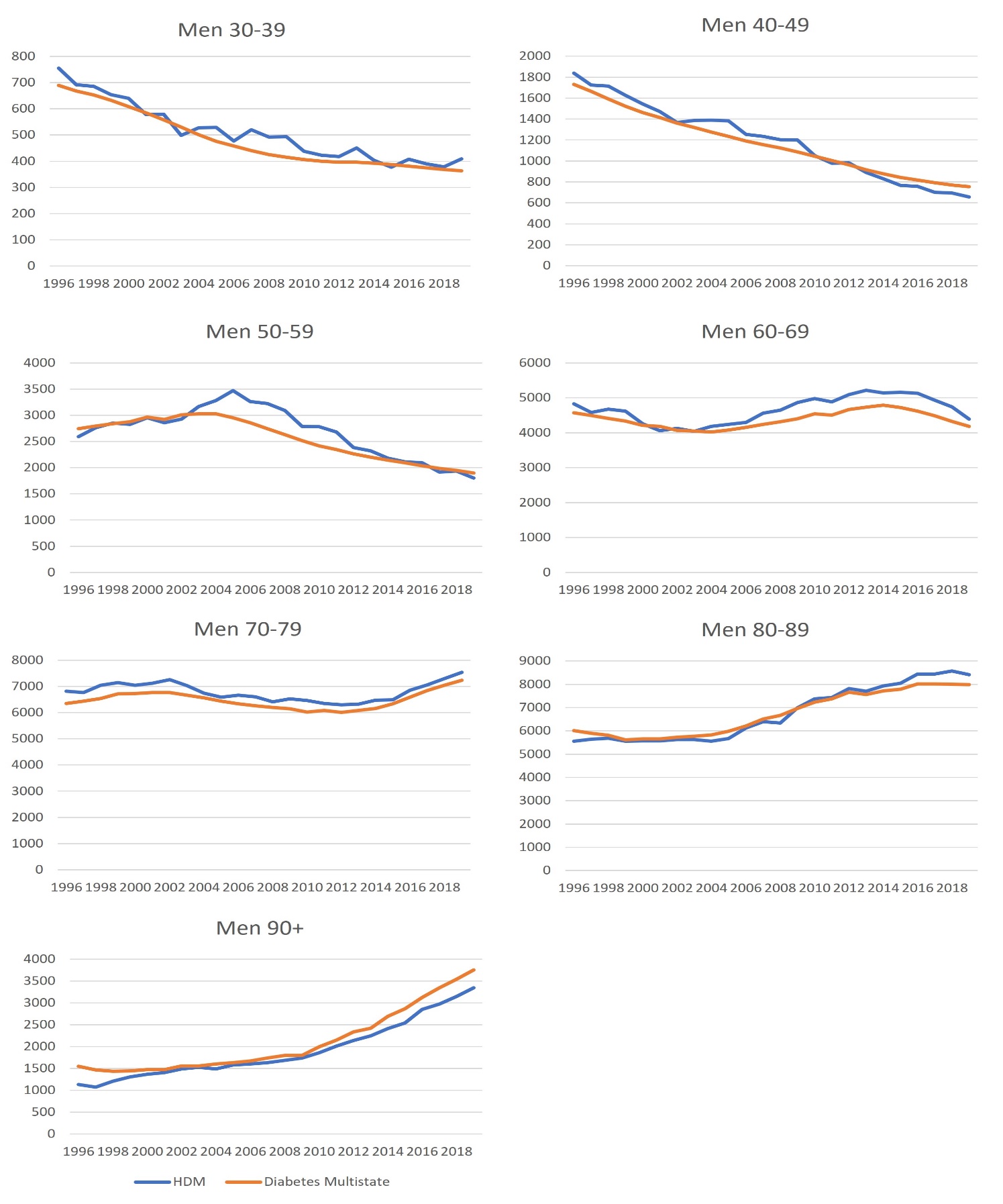
Figure S 7: predicted number of all-cause deaths against estimates from the Human Mortality Database between 1996-2019 for Finnish men by age group


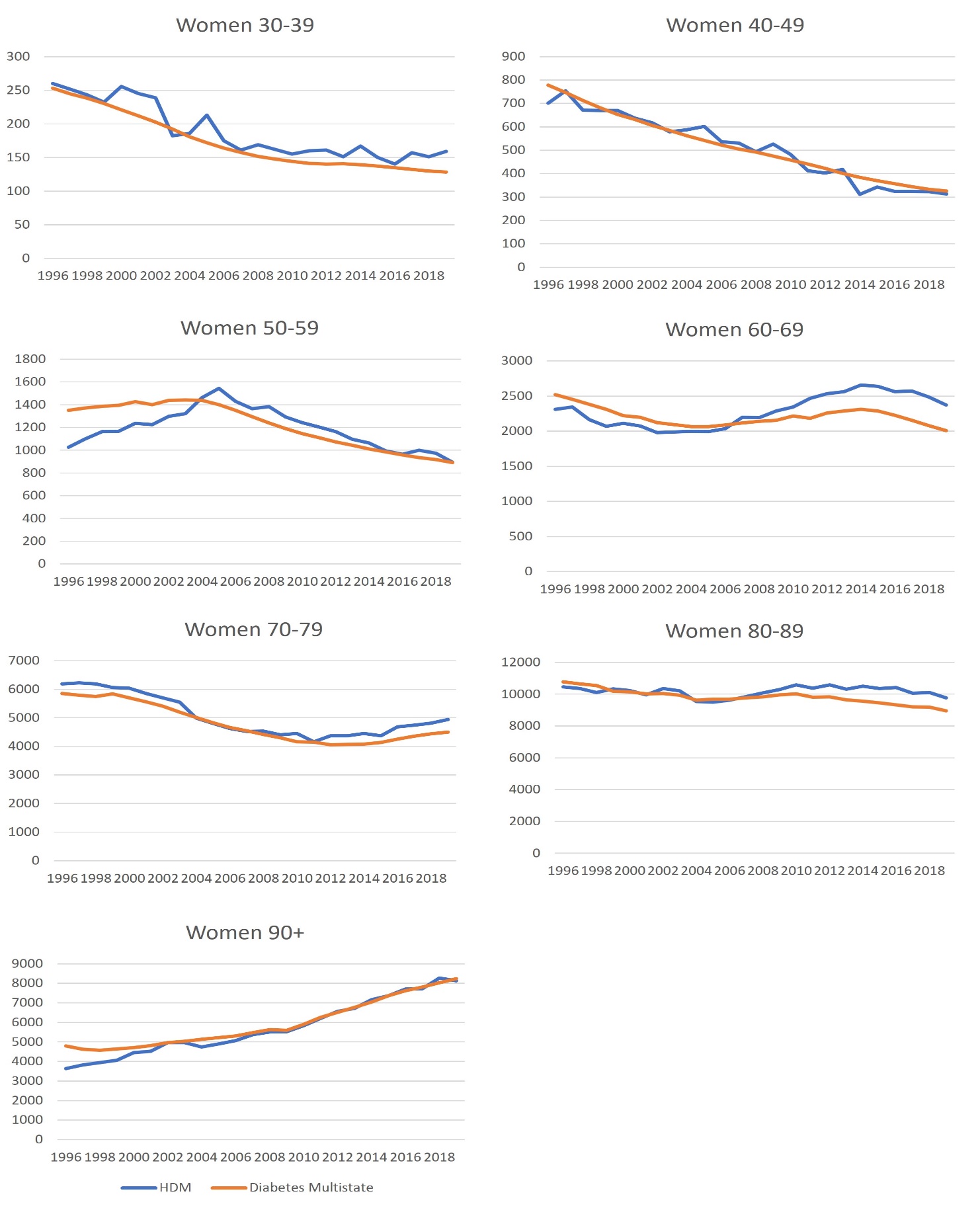


Figure S 8: predicted number of all-cause deaths against estimates from the Human Mortality Database between 1996-2019 for Finnish women by age group

# References

1. Niemi M, Winell K. Diabetes in Finland Prevalence and Variation in Quality of Care. Tampere; 2006.

2. Arffman M, Ilanne-Parikka P, Keskimäki I, Kurkela O, Lindström J, Sund R, et al. Tyypin 1 ja 2 diabeteksen ja niiden lisäsairauksien ilmaantuvuus ja esiintyvyys Suomessa vuosina 2000-2017. 2020;

3. Van Raalte A, Sasson I, Martikainen P. The case for monitoring life-span inequality. Science (80- ) [Internet]. 2018 Nov 30;362(6418):1002–4. Available from: https://doi.org/10.1126/science.aau5811

4. Koponen P, Borodulin K, Lundqvist, Annamari Sääksjärvi K, Koskinen S. Health, functional capacity and welfare in Finland – FinHealth 2017 study [Internet]. Helsinki; 2018. Available from: https://www.julkari.fi/bitstream/handle/10024/136223/Rap_4_2018_FinTerveys_verkko.pdf?sequence=1&isAllowed=y

5. Abdullah A, Peeters A, de Courten M, Stoelwinder J. The magnitude of association between overweight and obesity and the risk of diabetes: A meta-analysis of prospective cohort studies. Diabetes Res Clin Pract [Internet]. 2010;89(3):309–19. Available from: http://www.sciencedirect.com/science/article/pii/S0168822710001944

6. Flegal KM, Kit BK, Orpana H, Graubard BI. Association of All-Cause Mortality With Overweight and Obesity Using Standard Body Mass Index Categories: A Systematic Review and Meta-analysis. JAMA [Internet]. 2013 Jan 2;309(1):71–82. Available from: https://doi.org/10.1001/jama.2012.113905

**
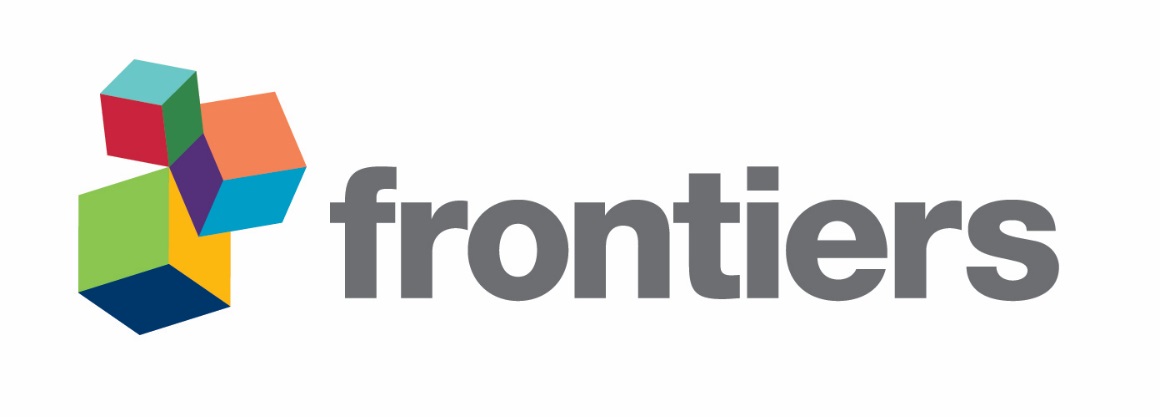
**
